# Investigating potential mediator between statin and coronary artery calcification

**DOI:** 10.1371/journal.pone.0203702

**Published:** 2018-09-18

**Authors:** Donghun Lee, Hyung Joon Joo, Ho-Won Jung, Do-Sun Lim

**Affiliations:** 1 Korea University Business School, Seoul, Korea; 2 Department of Cardiology, Cardiovascular Center, Korea University Anam Hospital, Seoul, Korea; Nagoya University, JAPAN

## Abstract

Statins are mainstay anti-lipidaemic treatments for preventing cardiovascular diseases but also known to increase coronary artery calcification (CAC). However, underlying relationship between statin and CAC is still unclear. This study explored the mediating role of five statin-related biochemical factors [i.e., low-density lipoprotein (LDL)-cholesterol, high-density lipoprotein (HDL)-cholesterol, triglyceride, glucose, and high sensitivity C-reactive protein levels]. Seoul Metabolic Syndrome cohort study includes 1370 participants suspected of metabolic syndrome. For causal mediation analysis, the dataset for 2016 including 847 participants with coronary computed tomography without any missing value were analysed using the *Mediation* package in R software. This study identified a causal mediation mechanism of HDL-cholesterol among the five biochemical factors. It implied that statin treatment increases the HDL-cholesterol level, leading to decreasing the probability of CAC score > 0. Estimated values of interest in HDL-cholesterol mediation were (1) average causal mediation effect, -0.011 with 95% CI [-0.025, -0.003], (2) average direct effect, 0.143 with 95% CI [0.074, 0.219], and total effect, 0.132 with 95% CI [0.063, 0.209]. Its mediation effect was maintained regardless of statin intensity. Sensitivity analysis also provided a robustness of the results under potential existence of a confounder between HDL-cholesterol and CAC. This study suggests a potential causal pathway between statin and CAC (the positive association of statin on CAC) through HDL-cholesterol as an inhibitor.

## Introduction

Statins are recommended as the drug of choice for dyslipidemia to prevent cardiovascular (CV) morbidity and mortality [1, 2]. The current guideline and evidence emphasized potent statin therapy [>50% reduction in serum low-density lipoprotein (LDL)-cholesterol level] for secondary prevention and expanded its use even for patients with moderate risks of CV diseases [3, 4]. The global market sales of statins were recorded to be up to $28.5 billion in 2014, and their sales figures are expected to increase continuously over time [5, 6]. Although the beneficial effect of statins has been thought to outweigh their side effects, several concerns, such as increased risks for diabetes mellitus and coronary artery calcification (CAC) development, remain.

CAC detected by computed tomography (CT) is a subclinical marker of coronary atherosclerosis [7, 8]. The extent of CAC was significantly correlated with total coronary plaque burden as well as CV diseases [9, 10]. Previously, Saremi et al. [11] reported that statin was associated with CAC progression in patients with type 2 diabetes mellitus. Henein et al. [12] also showed an accelerating effect of statin on CAC development. In contrast, the other studies have demonstrated that statin and the resulting lower serum LDL cholesterol level have an inverse relationship with CAC volume [13–15]. Thus, the relationship between statin and CAC still remains controversial.

Both patients taking statins and patients with CAC share many of the common CV risk factors. Previous studies have demonstrated that CAC was associated with age, sex, glucose level, high-density lipoprotein (HDL)-cholesterol level, LDL-cholesterol level, triglyceride level, body mass index (BMI), diabetes mellitus, and C-reactive protein (CRP) level [16–18]. In addition, statin use has been also associated with these risk factors [19, 20]. This now raises questions on the mechanism of the risk factors such as blood lipids and glucose level influenced by statin, resulting in CAC development. Furthermore, more confusions arise from the ambivalent effects of statin, such as CV protection and CAC development occurring at the same time.

The purpose of this study was to investigate the mediating role of each of five statin-related biochemical factors [LDL-cholesterol, HDL-cholesterol, triglyceride, glucose, and high-sensitivity (hs) CRP levels] in the association between statin and CAC. To that end, a set of direct and indirect pathways linking statin and CAC via a mediator was hypothesized. The total, direct, and mediation effects were statistically assessed using a mediation analysis.

## Methods

### Source of data

The Seoul Metabolic Syndrome cohort study led by Seoul Metropolitan Government has collected data of citizens suspected of metabolic syndrome from 2014 (Metabolic Syndrome Cohort in Korea, NCT02077530). A total of 1,370 participants were recruited from 25 public healthcare centres in Seoul between 2014 to 2015. The initial check-up and follow-up visit were performed in Korea University Anam Hospital. In the cohort, 870 participants took the annual follow-up visit and underwent coronary CT in 2016. The dataset includes self-administered questions about lifestyle, disease, and medication history as well as medical records including physical examination, laboratory measurement, and coronary CT. Finally, data of 847 participants (425 men and 422 women) after removing the subjects with missing values were analysed. The present study was approved by the institutional review board of Korea University Anam Hospital (IRB NO. ED13087) and performed in accordance with the principles of the Declaration of Helsinki. Written informed consent was also obtained.

### Measurements

Medication history including statins was confirmed by checking the prescriptions of the participants. Statin intensity was categorized in accordance with the 2013 ACC/AHA guideline [1]. Blood sample was obtained under at least an 8-hour fasting status: Serum glucose level was determined using a UV assay; Serum levels of total cholesterol, LDL cholesterol, HDL cholesterol, and triglycerides were measured using homogeneous enzymatic colorimetric assay; The hsCRP was gauged using immunoturbidimetry assay. Covariates (pre-treatment confounders) such as age, sex, SBP (systolic blood pressure), and smoking were controlled in the mediation analysis.

Coronary CT was performed using a second-generation dual-source CT scanner (Somatom Definition Flash; Siemens Healthcare, Forchheim, Germany) with a 2 × 128 × 0.6-mm^3^ section collimation and a 280 ms ration time. Initially, CT scanning was performed to evaluate the CAC score with 120-kV tube voltage, 80-mAs effective tube current, and 3-mm section thickness. The total estimated radiation dose for cardiac CT examinations ranged from 3 to 15 mSv. The CAC score was quantified on the basis of the Agatston scoring methods [21]. CAC as outcome variable was coded into binary 0 for zero CAC scores and 1 for positive scores.

### Statistical analysis

**Fig 1:**
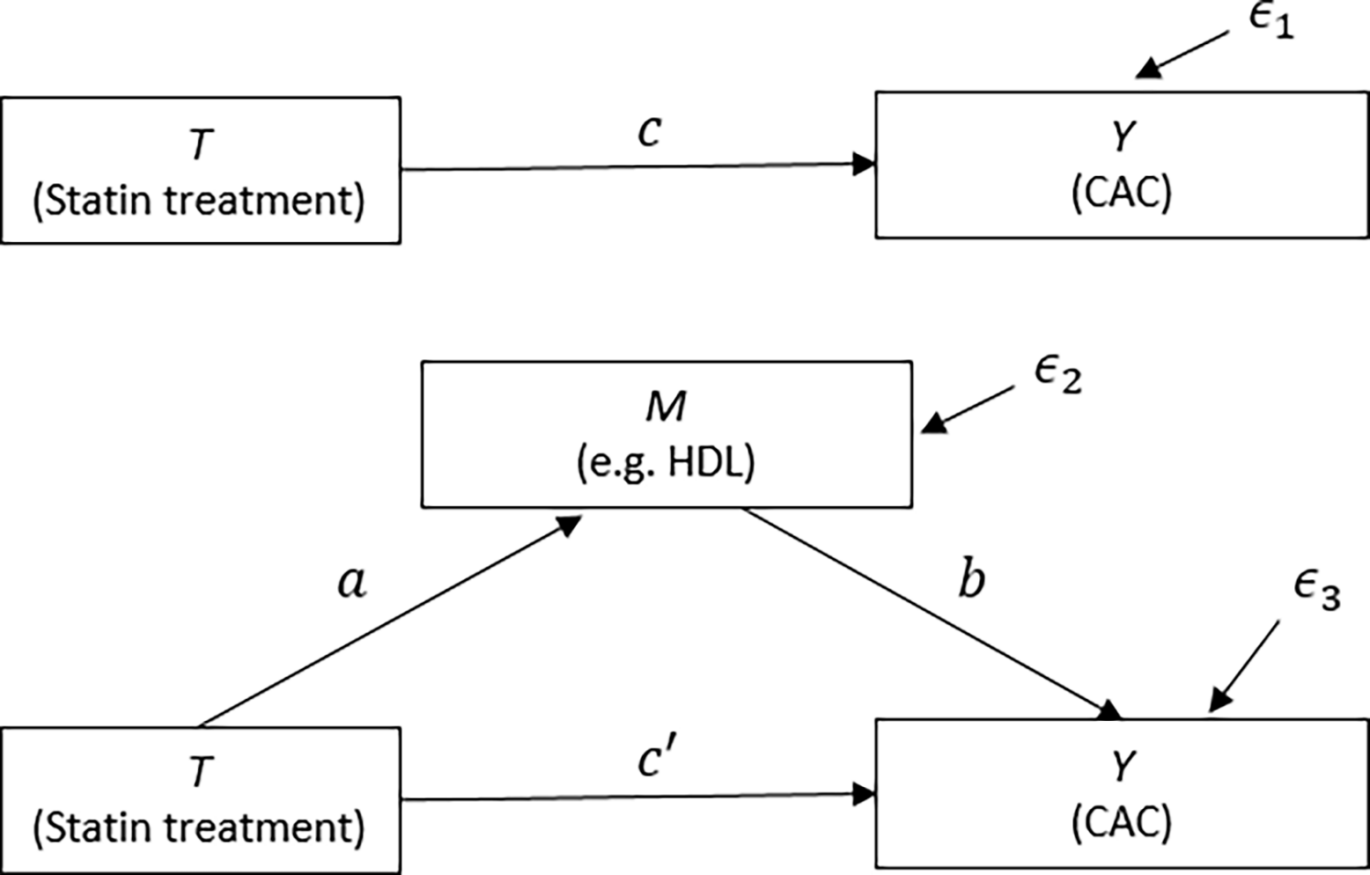
Causal mechanism diagram. Hypothesized direct and indirect pathways linking treatment assignment (T, e.g., statin) to outcome (Y, e.g., CAC) through a mediator (M, e.g., HDL-cholesterol). CAC, coronary artery calcification. *є*_*1*_, *є*_*2*_, and *є*_*3*_ means error terms.

Characteristics of the study population were described as mean and count for continuous and categorical variables, respectively. Associations (represented by *a*, *b* and *c* in Fig 1) between statin use and CAC with each of the potential mediators were examined using regressions with bootstrapping. The confidence intervals (CIs) were estimated via a bias-corrected bootstrapping with 1,000 replications, considering the skewed nature of the measurement such as CAC and hsCRP.

*R Mediation* package 4.4.6 [22] was adopted to perform the causal mediation analysis. The causal mechanism describes that treatment (statin) affects outcome (CAC) through an intermediate variable (e.g., HDL-cholesterol) decomposing total effect into direct effect (Statin → CAC) and indirect effect (Statin → Mediator → CAC) (Fig 1).

Causal mediation mechanism can be identified in the satisfaction of assumptions called “Sequential Ignorability” (SI) [23]:

- Treatment assignment (*T*) is assumed to be ignorable, i.e., statistically independent of the potential mediator (*M)* given the observed pre-treatment covariates (e.g., age, gender, and smoking). That is, no unmeasured common pre-treatment covariates affecting to *T* and *M*.
- Treatment assignment (*T*) is independent of potential outcomes (*Y*) given the observed pre-treatment covariates (confounders), i.e., no unmeasured common pre-treatment confounders affecting to *T* and *Y*.
- The mediator (*M*) is also ignorable given the actual treatment status and pre-treatment confounders, i.e., no unmeasured common pre-treatment confounders affecting to *M* and *Y*.

This study followed the model-based causal mediation analysis assuming the SI assumptions [22, 23]; the patients who have the same values of the pre-treatment covariates are regarded as if randomized, since the covariates were adjusted in analysis [24]. The mediator was modeled with a linear regression including treatment and pre-treatment covariates (i.e., age, gender, systolic blood pressure (SBP), and smoking). The outcome model was a logistic regression including the mediator, the treatment, and the pre-treatment covariates. These models were adjusted with the pre-treatment covariates.

Sensitivity analysis examines how the study results are robust to the violation of the last assumption above. Sensitivity parameter measures the correlation *ρ* between the two error terms *ϵ*_2_ and *ϵ*_3_ in the mediator (*M*) and outcome (*Y*) regression models. If the assumption holds, then the correlation has a value of 0. Data were analysed using R 3.4.0 software.

## Results

### Descriptive characteristics

Table 1 shows the demographic, laboratory, CAC, and statin treatment characteristics of the study sample (n = 847) comprising 50.2% men. Mean age was 56.69 (SD = 6.57) years old. A total of 202 subjects took statin: 86 men (23.8%) and 116 women (76.2%). Majority of them (89.1%) received moderate intensity statin treatment. Atorvastatin and rosuvastatin accounted for over 80% of the statins prescribed. The group taking statin (n = 202) had lower LDL cholesterol (p < .001), triglyceride (p = 0.006) levels and higher HDL-cholesterol (p = 0.001) and glucose (p < .001) levels than the group not taking statin (n = 645). Smoking, waist circumference, and BMI were similar in both groups. In addition, there was a high prevalence (37.6%; n = 76; p = 0.002) of CAC along with a high CAC score (80.27; SD = 237.91; p = 0.005) in the group who received statin therapy.

**Table 1:** Baseline characteristics.

	Total (n = 847)	Statin group (n = 202, 23.8%)	No statin group (n = 645, 76.2%)	P- value*
Age (years), mean (SD)	56.69 (6.57)	57.29 (5.87)	56.50 (6.77)	0.139
Men, n (%)	425 (50.2)	86 (42.6)	339 (52.6)	0.017
Current smoker, n (%)	153 (18.1)	39 (19.3)	114 (17.7)	0.673
Medication for diabetes mellitus, n (%)	88 (10.4)	46 (22.8)	42 (6.5)	< .001
Medication for hypertension, n (%)	209 (24.7)	93 (46.0)	116 (18.0)	< .001
Metabolic syndrome, n (%)	545 (64.3)	186 (92.1)	359 (55.7)	< .001
Body mass index (kg/m^2^), mean (SD)	25.81 (2.95)	25.92 (3.06)	25.78 (2.91)	0.569
Waist circumference (cm), mean (SD)	89.73 (7.69)	89.88 (7.68)	89.69 (7.70)	0.760
Blood pressure (mmHg), mean (SD)				
Systolic	127.60 (14.22)	125.95 (15.03)	128.12 (13.93)	0.057
Diastolic	84.33 (10.01)	82.53 (9.55)	84.89 (10.10)	0.003
Laboratory findings				
Total cholesterol (mg/dL), mean (SD)	191.18 (37.72)	163.70 (33.98)	199.78 (34.61)	< .001
HDL-cholesterol (mg/dL), mean (SD)	49.94 (12.34)	52.50 (11.77)	49.13 (12.42)	0.001
LDL-cholesterol (mg/dL), mean (SD)	127.15 (37.71)	98.43 (33.13)	136.14 (34.44)	< .001
Triglycerides (mg/dL), mean (SD)	168.98 (125.30)	147.78 (86.62)	175.62 (134.53)	0.006
Fasting glucose (mg/dL), mean (SD)	101.77 (22.45)	106.90 (32.40)	100.16 (17.99)	< .001
hsCRP (mg/dL), mean (SD)	1.24 (2.51)	1.00 (1.63)	1.31 (2.73)	0.129
CAC				
CAC, mean (SD)	46.87 (192.94)	80.27 (237.91)	36.42 (175.44)	0.005
CAC > 0, n (%)	242 (28.6)	76 (37.6)	166 (25.7)	0.002
Statin intensity, n (%)				
Low		10 (5.0)		
Moderate		180 (89.1)		
High		12 (5.9)		
Statins, n (%)				
Atorvastatin		92 (45.5)		
Fluvastatin		2 (1.0)		
Pitavastatin		15 (7.4)		
Pravastatin		5 (2.5)		
Rosuvastatin		77 (38.1)		
Simvastatin		11 (5.4)		

SD, standard deviation; HDL, high-density lipoprotein; LDL, low-density lipoprotein; CAC, coronary artery calcification; hsCRP, high-sensitivity C-reactive protein; p, p-value for the group taking statin and the group not taking statin.

*, χ^2^ or *t*-test results.

### Associations between statin, potential mediators, and CAC

After adjustment of the covariates, regression analysis showed the association between statin, potential mediators, and CAC (Table 2).

**Table 2:** Associations between statin, potential mediators, and CAC.

Mediator	Equation	Path	Path coefficient	p	95% CI
	CAC = *c* Statin + *є*_*1*_	*c*	2.006	< .001	[1.383, 2.951]
LDL-cholesterol	LDL-cholesterol = *a* Statin + *є*_*2*_	*a*	-37.906	< .001	[-43.072, -32.734]
	CAC = *c´* Statin + *b* LDL-cholesterol + *є*_*3*_	*b*	0.999	0.747	[0.994, 1.004]
HDL-cholesterol	HDL-cholesterol = *a* Statin + *є*_*2*_	*a*	2.993	0.002	[1.168, 4.993]
	CAC = *c*´ Statin + *b* HDL-cholesterol + *є*_*3*_	*b*	0.981	0.008	[0.968, 0.995]
Triglyceride	Triglyceride = *a* Statin + *є*_*2*_	*a*	-21.392	0.030	[-35.796, -6.302]
	CAC = *c*´ Statin + *b* Triglyceride + *є*_*3*_	*b*	1.001	0.102	[0.999, 1.002]
Glucose	Glucose = *a* Statin + *є*_*2*_	*a*	7.399	< .001	[3.194, 12.447]
	CAC = *c*´ Statin + *b* Glucose + *є*_*3*_	*b*	1.002	0.555	[0.995, 1.012]
hsCRP	hsCRP = *a* Statin + *є*_*2*_	*a*	-0.314	0.124	[-0.644, < .001]
	CAC = *c*´ Statin + *b* hsCRP + *є*_*3*_	*b*	0.958	0.378	[0.824, 1.027]

$\hat{\beta }$, coefficient from the study sample; CI, confidence interval from a bias-corrected bootstrapping with 1,000 replications; CAC, coronary artery calcification (outcome variable 0 for zero scores and 1 for positive scores); Statin, statin use (independent variable); LDL, low-density lipoprotein; HDL, high-density lipoprotein; hsCRP, high-sensitivity C-reactive protein.

*c*, odds ratio from a logistic regression between statin use and CAC; *a*, coefficient from a linear regression between each of potential mediators and the independent variable; *b*, odds ratio from a logistic regression between the mediator and the outcome variable, controlled for the independent variable; *є*_*1*_, error term (*i* = 1, 2, 3).

All the analysis were adjusted for age, sex, systolic blood pressure, and smoking.

Path *c*: Statin had a positive association with the CAC (odds ratio 2.006; p < .001; 95% CI: 1.383 to 2.951), suggesting that the subjects with statin treatment had higher odds of positive CAC scores than those without statin treatment.

Path *a*: Statin was associated with the lower LDL-cholesterol (-37, 906; p < .001; 95% CI: -43.072 to -32.734) and triglyceride (-21.392; p < .030; 95% CI: -35.796 to -6.302) levels, and was associated with the higher HDL-cholesterol (2.993; p = 0.002; 95% CI: 1.168 to 4.993) and glucose (7.399; p < .001; 95% CI: 3.194 to 12.447) levels. On the other hand, no significant association was found between statin use and the hsCRP level.

Path *b*: HDL-cholesterol level was significantly associated with a lower prevalence of CAC (odd ratio 0.981; p = 0.008; 95% CI: 0.968 to 0.995). In the other hand, no significant relationships were observed between the remaining mediators and CAC.

### Identification of causal mediation mechanism between statin and CAC

Causal mediation analysis with each of the five biochemical factors as a mediator (Fig 1) was performed with the options of a nonparametric 1,000 bootstrapping replicates and 95% CI, while adjusting for the covariates. Table 3 shows the point estimates and 95% CIs of total, direct, and mediation effects from each mediation model. Direct and mediation effects are called average direct effect (ADE) and average causal mediation effect (ACME, also refer to as indirect effect). Statistically, total effect is the sum of ACME and ADE. The ACME states whether statin use (treatment) positively (the probability of increasing CAC) or negatively (the one of decreasing CAC) affects outcomes (CAC) through a mediator (e.g., HDL-cholesterol). Thus, if the upper limit of bootstrap CI is less than zero, then our hypothesis of negative causal mediation effect (i.e., H_0_ = 0, H_1_: ACME < 0) is supported at the one-tailed level of α = 0.025. On the other hand, if the lower limit of bootstrap CI is greater than zero, then our hypothesis of positive causal mediation effect, H_1_: ACME > 0, cannot be rejected at the one-tailed level of α = 0.025.

**Table 3:** ACME, ADE, and total effect and their 95%CIs between statin and CAC.

Mediator	ACME	ADE	Total effect
LDL-cholesterol	0.006 [-0.027, 0.042]	0.127 [0.053, 0.213]	0.132 [0.061, 0.208]
HDL-cholesterol	-0.011 [-0.025, -0.003]	0.143 [0.074, 0.219]	0.132 [0.063, 0.209]
Triglyceride	-0.004 [-0.010, 0.005]	0.137 [0.067, 0.214]	0.133 [0.061, 0.207]
Glucose	0.003 [-0.008, 0.017]	0.129 [0.056, 0.207]	0.132 [0.063, 0.208]
hsCRP	0.003 [-0.001, 0.020]	0.130 [0.061, 0.204]	0.133 [0.064, 0.209]

CAC, coronary artery calcification; LDL, low-density lipoprotein; HDL, high-density lipoprotein; hsCRP, high-sensitivity C-reactive protein; ACME, average causal mediation effect; ADE, average direct effect. The confidence intervals (CIs) in brackets are 95% CIs from a 1,000 nonparametric bootstrap replicates. All the analysis were adjusted for age, sex, systolic blood pressure, and smoking.

Total effect and ADE (Table 3) show that statin is likely to increase the probability of CAC > 0 in all five mediation models. This result is consistent with previous studies [14, 25]. Importantly, ACME proposed only HDL-cholesterol as a significant mediator mitigating statin-induced increased probability of CAC > 0. It implies that statin increases HDL-cholesterol level, which in turn makes the subject less likely to have CAC > 0 (note that CAC was analysed as a binary variable of CAC score = 0 and CAC score > 0). Any other factors failed to show mediation effect between statin and CAC.

One measure of interest is the proportion of mediation defined as the proportion of total effect explained by the mediator. Average proportion of the HDL-cholesterol driven mediation effect becomes 8.3% (calculated as the percentage of ACME/total effect [0.011/0.132 X 100]). The total effect implies that statin increased the percentage chance of CAC > 0 by 13.2% in HDL-cholesterol causal mediation model. Other figures can be interpreted in the same context.

### Robustness of the causal mediation effect

The robustness of the causal mediation effect of HDL-cholesterol was explored by (1) the additional analyses of three different datasets according to statin intensity and (2) sensitivity analysis.

The study population had 202 subjects treated with statin, and they can be divided into the 3 different subgroups according to statin intensity: low-intensity statin treatment group (10 patients), moderate-intensity statin treatment group (180 patients), and high-intensity statin treatment group (12 patients). Additional causal mediation analyses were performed with the 3 different datasets formed as follows:

- Dataset 1 (n = 837): excluding the 10 subjects with low-intensity statin treatment
- Dataset 2 (n = 825): excluding the 22 subjects with low and high-intensity statin treatment
- Dataset 3 (n = 835): excluding the 12 subject with high-intensity statin treatment

The ACME with HDL-cholesterol mediator was estimated by applying the nonparametric bootstrapping method: ACME = -0.011 with 95% CI [-0.025, -0.003] for dataset 1; ACME = -0.011 with 95% CI [-0.027, -0.003] for dataset 2; and ACME = -0.010 with 95% CI [-0.026, -0.002] for dataset 3. These results were consistent with that of total population, and suggested that the mediation effect of HDL-cholesterol between statin and CAC might be independent to statin intensity.

**Fig 2:**
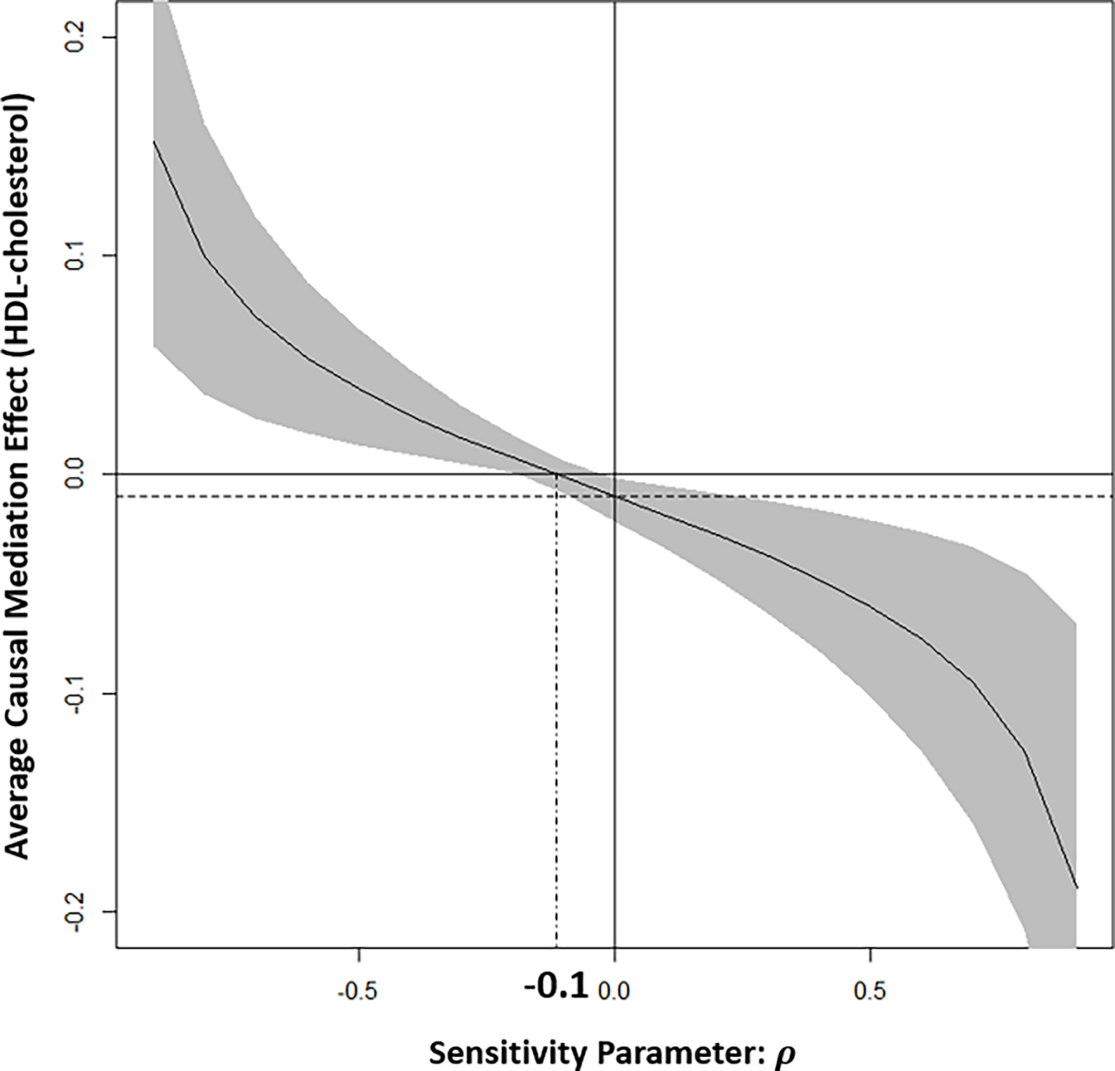
Sensitivity analysis of the ACME of HDL-cholesterol.

This study performed sensitivity analysis to investigate the robustness of HDL-cholesterol mediation model when an unobserved confounder between HDL-cholesterol and CAC was considered. Fig 2 shows the ACMEs of HDL-cholesterol against varying sensitivity parameter ρ (the correlation between two error terms in ϵ2 and ϵ3) and 95% CIs represented by the shaded region. When the sensitivity parameter has a value in −0.1 ≤ *ρ* ≤ 1, the ACME of HDL-cholesterol could be identified. On the other hand, the sensitivity parameter ρ is less than -0.1, the ACME of HDL-cholesterol would have a positive value contrary to our study results (negative point estimate of ACME, -0.011 in Table 3). The vertical dashed line represent the point of ρ when the ACME becomes zero.

## Discussion

To the best of our knowledge, this is the first study that explored the mediating role of the five statin-related biochemical factors in the relationship between statin and CAC. The main findings of the present study are as follows: (1) when adjusting for the covariates (age, sex, SBP, and smoking), statin use was positively associated with CAC (odds ratio 2.006; p < .001; 95% CI: 1.383 to 2.851 in Table 2). In addition, the direct effects of statin on CAC were consistently positive, even when the each mediation effects of five statin-related biochemical factors (LDL-cholesterol, HDL-cholesterol, triglyceride, fasting glucose, and hsCRP) was excluded (see also ADEs (direct effects) in Table 3). (2) The total effects between statin and CAC were mainly explained by the direct effects (see the magnitudes of total and direct effects in Table 3). (3) Among the five biochemical factors, only HDL-cholesterol was revealed as a significant mediator with a negative mediation effect value of -0.011 with 95% CI [-0.025, -0.003] even after adjusting for covariates and its direct effect. It indicates that HDL cholesterol level affected by statin treatment could make the subject less likely to have CAC > 0.

Coronary calcium formation is an integral part of the atherosclerotic process. CAC score is closely correlated with the overall coronary artery disease severity and its clinical outcomes [26, 27]. Although the beneficial effects of statin on coronary artery plaque progression or cardiovascular clinical outcomes have been clearly established [28, 29], recent coronary CT data have shown that statin significantly increases CAC development, which was called as the “statin paradox” or the “plaque paradox” [12, 25]. Moreover, invasive intravascular imaging data also demonstrated that statin increased coronary calcification [30, 31]. Interestingly, it was accompanied with an overall plaque regression, suggesting that statin-induced coronary calcification represents plaque repair rather than plaque progression. However, there have been insufficient data regarding whether coronary calcification could be an indicator of the stability of an atherosclerotic plaque [32].

Despite recent studies showing the “statin paradox”, previous in vitro study results supported the inhibitory effect of statin use on vascular calcification [33–35]. Son et al. revealed that atorvastatin attenuated the phosphate-induced calcification process of the vascular smooth muscle cell via the Gas6/Axl pathway [34]. In addition, statins have anti-inflammatory effects. For example, statin decreased hsCRP level, regardless of the LDL cholesterol level [36]. Statins also decreased the amount of proinflammatory cytokines secreted from the endothelial cells and macrophage [37, 38]. Thus, these controversial results elicit questions on the direct and indirect effects of statin use on vascular calcification.

A previous community-based study showed that HDL-cholesterol level was significantly associated with CAC [39]. HDL-cholesterol was also reported to inhibit the calcification of vascular cells via anti-osteogenic differentiation and inflammatory cytokine reduction (interleukin-1β and 6) [40]. These results support our result that the HDL-cholesterol might play a mediator, which attenuates statin-induced CAC development. The mediation analysis that the present study adopted might provide new insights on the underlying mechanisms between statin and CAC development.

Although the present study demonstrated statin-mediated higher HDL-cholesterol could contribute to the attenuation of coronary artery calcification, other lipid-independent mechanisms for vascular calcification should be considered as well. For example, cathepsin signaling has been studied to be associated with vascular calcification process [41]. In addition, statin treatment lowered plasma and tissue cathepsin levels [42, 43]. Thus, these data imply that cathepsin signaling could play an important role in statin-associated coronary artery calcification development and progression regardless of lipid profile. Further research to investigate the novel mechanisms for vascular calcification remains to be conducted.

There are several considerations in causal mediation analysis and its interpretation. First, the present study was cross-sectional, and the treatment (statin use) was not randomly assigned. There exists a threat in interpreting our results in causal terms. However, the cross-sectional data might have potential to address “research questions of a longitudinal nature” [44]. This approach could be more important for the ethically impermissible researches with random mediation assignment or potential hazardous intervention. Second, the present study performed the sensitivity analysis in terms of the “Sequential Ignorability” assumption. The more critical the sensitivity parameter, the more the robustness. However, it is uncertain how robust is robust enough to conclude valid (or robust) results because there is no objective criterion to determine whether the assumption between mediator and outcome is valid [24]. Sensitivity analysis can be used to assess the robustness of one conclusion relative to those other similar studies [24]. Third, prevalence of CAC in the study population was relatively high (approximately 29%, n = 242). The use of logistic regression may lead to overestimate the odds ratios [45]. However, a negative mediation effect of the HDL-cholesterol with a value of -0.028 (95% CI: -0.077 to -0.002) was identified under the same mediation analysis with a linear regression of natural log of CAC score + 1 (ln(CAC + 1)) [46]. In addition, for the dataset (n = 845) excluding outliers in the mediator and outcome models (e.g., logistic regression including statin use, HDL-cholesterol, CAC and covariates) of the causal mediation model based on the studentized residuals [47], we also identified a negative mediation effect of the HDL-cholesterol with a value of ACME, -0.009 (95% CI; -0.023 to -0.002). Fourth, we only evaluated coronary artery calcium deposits. Extra-coronary vascular calcification has been also reported to be associated with cardiovascular morbidity and mortality [48, 49]. Thus, the results of other vascular sites, including aorta, carotid and peripheral arteries, might validate and generalize the current hypothesis.

## Conclusion

In conclusion, among five statin-affecting biochemical factors (HDL-cholesterol, LDL-cholesterol, triglyceride, glucose, and hsCRP levels), HDL-cholesterol may play an inhibitory mediating role in the potential causal pathway between statin and CAC development.

## Supporting information

S1 File Raw data. (XLS)
